# Discrete-state models identify pathway specific B cell states across diseases and infections at single-cell resolution

**DOI:** 10.1016/j.jtbi.2024.111769

**Published:** 2024-02-28

**Authors:** George Kassis, Mukta G. Palshikar, Shannon P. Hilchey, Martin S. Zand, Juilee Thakar

**Affiliations:** a Department of Microbiology and Immunology, University of Rochester School of Medicine and Dentistry, Rochester, USA; b Biophysics, Structural, and Computational Biology Program, University of Rochester School of Medicine and Dentistry, Rochester, USA; c Department of Medicine, Division of Nephrology, University of Rochester Medical Center, Rochester, NY, USA; d Department of Biostatistics and Computational Biology, University of Rochester School of Medicine and Dentistry, Rochester, USA; e Department of Biomedical Genetics, University of Rochester School of Medicine and Dentistry, Rochester, USA

**Keywords:** Single-cell RNA sequencing, Oxygen regulated signaling cascades, Boolean networks, Steady state analysis

## Abstract

Oxygen (O_2_) regulated pathways modulate B cell activation, migration and proliferation during infection, vaccination, and other diseases. Modeling these pathways in health and disease is critical to understand B cell states and ways to mediate them. To characterize B cells by their activation of O_2_ regulated pathways we develop pathway specific discrete state models using previously published single-cell RNA-sequencing (scRNA-seq) datasets from isolated B cells. Specifically, Single Cell Boolean Omics Network Invariant-Time Analysis (scBONITA) was used to infer logic gates for known pathway topologies. The simplest inferred set of logic gates that maximized the number of “OR” interactions between genes was used to simulate B cell networks involved in oxygen sensing until they reached steady network states (attractors). By focusing on the attractors that best represented sequenced cells, we identified genes critical in determining pathway specific cellular states that corresponded to diseased and healthy B cell phenotypes. Specifically, we investigate the transendothelial migration, regulation of actin cytoskeleton, HIF1A, and Citrate Cycle pathways. Our analysis revealed attractors that resembled the state of B cell exhaustion in HIV+ patients as well as attractors that promoted anerobic metabolism, angiogenesis, and tumorigenesis in breast cancer patients, which were eliminated after neoadjuvant chemotherapy (NACT). Finally, we investigated the attractors to which the Azimuth-annotated B cells mapped and found that attractors resembling B cells from HIV+ patients encompassed a significantly larger number of atypical memory B cells than HIV− attractors. Meanwhile, attractors resembling B cells from breast cancer patients post NACT encompassed a reduced number of atypical memory B cells compared to pre-NACT attractors.

## 1. Introduction

B cells play an important role in the immune response to infection, vaccination and diseases. During adaptive responses, extrafollicular B cells differentiate into either short-lived antibody secreting cells (ASCs) or early unmutated germinal center (GC)-independent somatically mutated memory B cells (MBCs). Alternatively, B cells with high enough affinity for antigens can make another fate decision by entering into germinal center reactions to differentiate into long-lived plasma cells (LLPCs), driving long-term immunity (Akkaya et al., 2020; Palm and Henry, 2019).

Despite our knowledge of B cell development and differentiation, there is still a significant gap in our understanding of how those processes respond to differing O_2_ levels during disease and infection (Thakar et al., 2020; Qiu et al., 2015). Specifically, O_2_ levels vary as B cells migrate from the relatively high O_2_ environment of the circulatory system to the low O_2_ environment of secondary lymphatic organs, which include highly hypoxic GCs (Carreau et al., 2011; Liu et al., 2011). B cells modulate their development within these varied O_2_ environments, in part, through factors such as HIF-1α (Hilchey et al., 2022, 2020). While B cell differentiation often depends on T cell help and CD40 signaling, B cell receptor (BCR) signaling can drive differentiation independent of T cells, regulated by additional factors such as NF-κB, PI3K/AKT, and KRAS, as reviewed in Laidlaw and Cyster (2021). Given the large number of interacting factors that regulate B cell development and activation, computational models prove very useful in resolving the complex gene and environmental interactions, and dynamically simulating them to obtain a better understanding of the underlying systemic mechanisms and behaviors (Walsh et al., 2011).

For many applications, quantitative kinetic models, which are based on differential equations, provide extraordinary insights into the function of biological systems (Resat et al., 2009). Nonetheless, these models require extensive information about structure and kinematic parameters that are often very difficult to obtain. On the other hand, Boolean networks are qualitative logic-based models that simulate the dynamic behavior of the systems while using minimal information. In these networks, genes are assumed to have a binary state: a gene is ON (1) if its concentration is above a threshold of activation and OFF (0) if its concentration is below that threshold (Thakar et al., 2012, 2007). Regulatory relationships between these genes are then modeled as logical statements that use the operators AND, OR, and NOT to calculate the states of genes based on the states of their upstream genes.

Previous approaches have used literature curation to define Boolean regulatory rules. We have previously shown that Boolean networks can be learned from large-scale omics datasets, including single-cell and bulk RNA sequencing, which provide a wealth of signaling information (Palli et al., 2020, 2019; Palshikar et al., 2023, 2022). Specifically, by analyzing the expression profile of each cell in a population separately, scRNA-seq provides insights into cellular heterogeneity in normal and diseased tissues and uncovers mappings to distinct cells within a population that might have different functions, histories, or stochastic alterations. Finally, the dynamic performance of inferred models facilitates the computation of their long-term behavior by highlighting steady states also referred to as attractors (Kauffman, 1969), corresponding to biologically relevant phenotypes.

Boolean network models have provided substantial biological insights (Krumsiek et al., 2011; Saadatpour et al., 2011). Building upon the concepts of Boolean networks, in this paper, we used the inference algorithm scBONITA (Palshikar et al., 2022) trained on publicly available scRNA-seq datasets of isolated B cells in order to investigate pathway specific B cell states. scBONITA infers logic rules that depict signal integration in convergent interactions. Here we show for the first time that the logic rules can be optimized using scRNA-seq data and that the pathway specific B cell attractors have overlapping states across multiple diseases and infections. Furthermore, scBONITA holds the advantage of developing discrete state network models from cross-sectional data. Cross-sectional data is more common in human immunology and other translational investigations because of the cost associated with participant visits at multiple time points. It is also typically easier to recruit participants for investigations with cross-sectional experimental design. We then simulated these network models to identify reachable steady signaling states or attractors, which corresponded to healthy and diseased cellular phenotypes as well as distinct B cell subtypes.

## 2. Methods

### 2.1. Data collection and processing

scRNA-seq data of isolated B cells was obtained for five studies from the Gene Expression Omnibus (GEO) repository (Edgar, 2002). The isolated B cells were obtained from healthy patients, Lung Cancer infiltrated tissue (GSE84789, SmartSeq2), Breast cancer infiltrated tissue (GSE135710, 10x Genomics), HIV patients (GSE157966, 10x Genomics), patients infected with SARS-CoV-2 (GSE164381, 10x Genomics), as well as patients that were hospitalized due to a SARS-CoV- 2 infection and either improved or succumbed to their disease (GSE158038, 10x Genomics). These datasets were preprocessed using the Seurat toolkit implemented in R (Butler et al., 2018; Hao et al., 2021; Satija et al., 2015; Stuart et al., 2019). Primarily, low quality cells or empty droplets were filtered out by removing any cells with feature counts of less than 200. Cell doublets were also filtered out by removing cells that had RNA counts of more than 2500. Cells that contained more than 5% of mitochondrial counts were removed.

Following the filtering of cells, the counts of each cell within each dataset were normalized by the total expression, multiplied by a scale factor of 10,000, and log2 transformed. It is worth noting that within each dataset, cells obtained from different subjects were separately filtered and normalized prior to the integration of subjects within each dataset. In preparation for integration, Seurat was used to find highly variable genes within each subject’s expression data, and genes that were repeatedly variable across subjects were identified for each of the five datasets. Then, in order to correct for technical noise and prepare each dataset for comparative scRNA-seq analysis, the repeatedly variable genes were used to integrate samples obtained from different subjects (Haghverdi et al., 2018). Each integrated dataset was inspected to ensure that the cells do not cluster based on the subjects. Following this inspection, any further analyses were performed on the normalized and integrated datasets.

### 2.2. Selection of networks

Nineteen prior knowledge networks related to cell B cell migration, proliferation, and differentiation (Table 1) were obtained from the Kyoto Encyclopedia of Genes and Genomes (KEGG) (Kanehisa, 2019, 2000; Kanehisa et al., 2021) or the WikiPathways database (Martens et al., 2021) and downloaded using the WikiNetworks Python package (Palshikar et al., 2022). scBONITA regulatory rule inference was performed using these networks’ topologies for each of the five datasets.

### 2 3. Application of scBONITA and attractor analysis

In this work, we used our previously published algorithm scBONITA (Palshikaret al., 2022a; Hilchey, 2022, freely available at https://github.com/Thakar-Lab/scBONITA) to infer Boolean logic rules for 19 signaling networks obtained from KEGG (cite KEGG) (Table 1). We briefly describe the scBONITA workflow below.

**Fig. 1.**
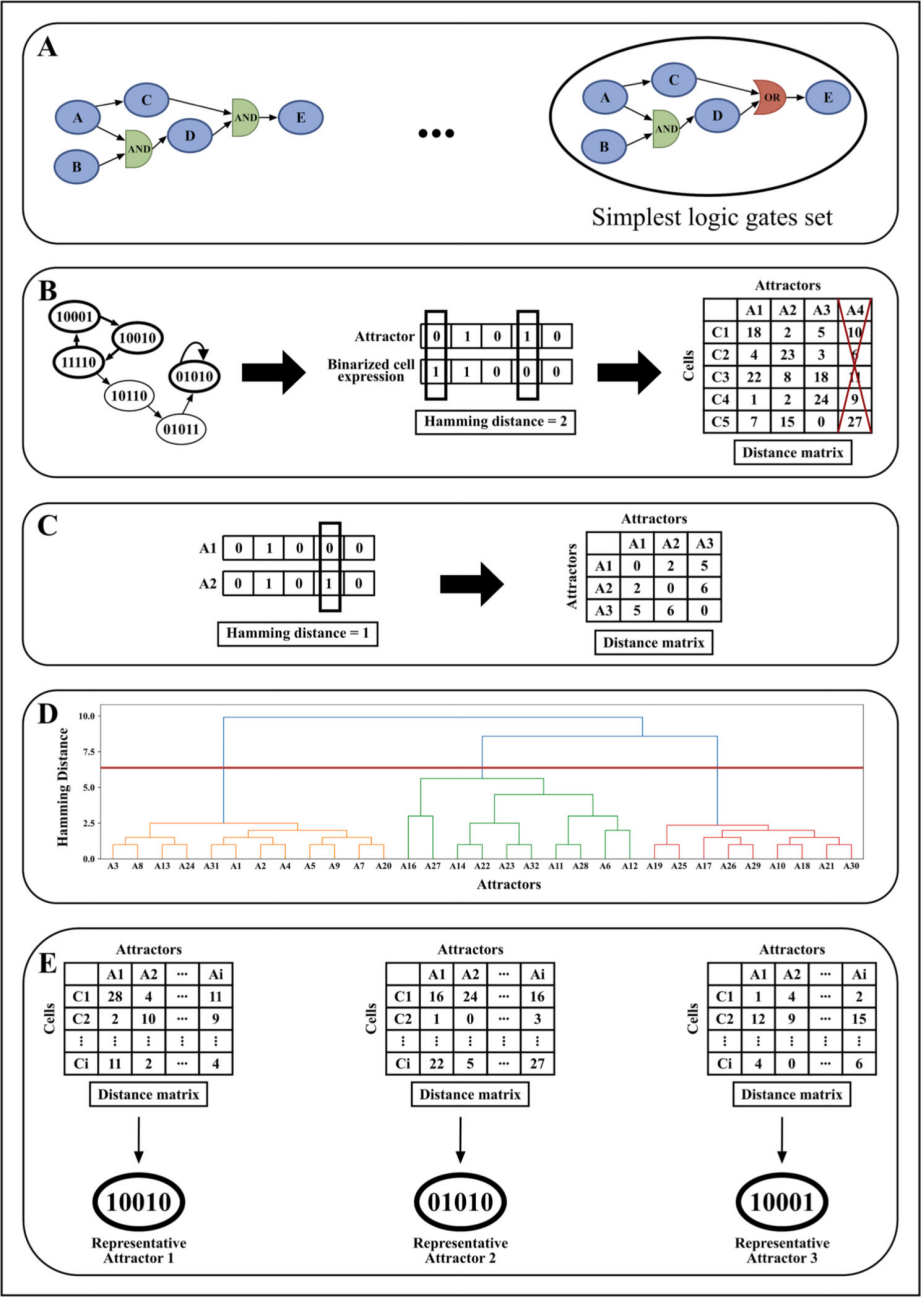
A summary of the methods used to identify and filter attractors. (A) The topology of a network as well as scRNA-seq data are provided as input into scBONITA, which then outputs several equally optimized rule sets, out of which we chose the simplest rule set that maximizes OR rules. (B) The chosen rule set was synchronously simulated starting from the binarized expression states of sequenced cells until a steady state (attractor) was reached. Hamming distance was calculated between each attractor reached and each sequenced cell. Attractors that did not have the smallest Hamming distance to at least one cell were filtered out. (C) Hamming distance was calculated between each remaining attractor to generate a distance matrix. (D) The distance matrix was fed into a hierarchical agglomerative clustering algorithm, where the threshold that divided clusters (red line) corresponded to 20% of the number of nodes in the analyzed network. (E) Withing each cluster, Hamming distances between attractors and each cell in the dataset were calculated. The attractor that had the smallest Hamming distance to the highest number of cells was used to represent the entire cluster.

1. Binarization of normalized count matrix: scBONITA binarizes the count matrix based on a user-defined threshold (here, 0.001), to classify genes as either ON or OFF in accordance with the Boolean network framework. We selected this threshold based on the observed distributions of normalized counts in our test datasets (Fig. A.1). Binarized data is used for the following steps. Note that Initiating Boolean models with the observed state reduces the time to reach optimized solution by the genetic algorithm. By definition, when the state of the system does not change it is considered as a fixed attractor or steady state. The goal of our approach is to identify the logic rules underlying the observed state, which is assumed to be a steady state.
2. Boolean rule inference: scBONITA uses a combination of a genetic algorithm and an exhaustive local search to score candidate rules to identify equivalent rule sets (ERS) which explain the observed data equally well. The ERS consists of one or more best-scoring Boolean rules per node in the network. These rule sets can be used to simulate and perturb the network to perform attractor analysis and pathway analysis. Here, we use the attractor analysis functionality of scBONITA.
3. Attractor analysis:
  1. Selection of minimal rule sets: From the ERS defined above, scBONITA selects minimal rule sets to simulate the networks. These minimal rule sets maximize the ‘OR’ terms in the Boolean function (Fig. 1A) If optimized Boolean models have the same number of ‘OR’ gates, one model is chosen randomly.
  2. Network simulation: The networks were simulated using these minimal rule sets. The observed vectors of gene expression per cell were selected as start states and the network was simulated until attractors, or stable recurring states, were reached. These attractors have been shown to correspond to biologically relevant phenotypes; intuitively, steady signaling states correspond to stable phenotypes.
  3. Cells-to-attractors mapping: For each network, we calculated the Hamming distances between each observed cell and the calculated attractors. Each cell was assigned to one attractor per network which had the lowest Hamming distance to its binarized expression vector. Attractors that were not assigned to any of the cells were discarded (Fig. 1B).
  4. Merging of similar attractors: Attractors were clustered using agglomerative hierarchical clustering, using the Hamming distance between them. Attractors with a Hamming distance of ≤20% of the size of the network were merged. Gene-level conflicts within the merged attractors were resolved by assigning an ‘on’ or ‘1’ value to that gene (Fig. 1C and D).
  5. Attractors to cell states: The attractor with the maximum number of mapped cells within an attractor cluster was used to represent a pathway-specific cell state (Fig. 1E). Finally, in order to determine if the representative attractors and source of B cells (healthy or diseased/infected patients) were related, we performed Chi- squared tests of independence for each combination of network and dataset that was optimized by scBONITA (Table A.1).

### 2.4. B cell annotations

**Fig. 2.**
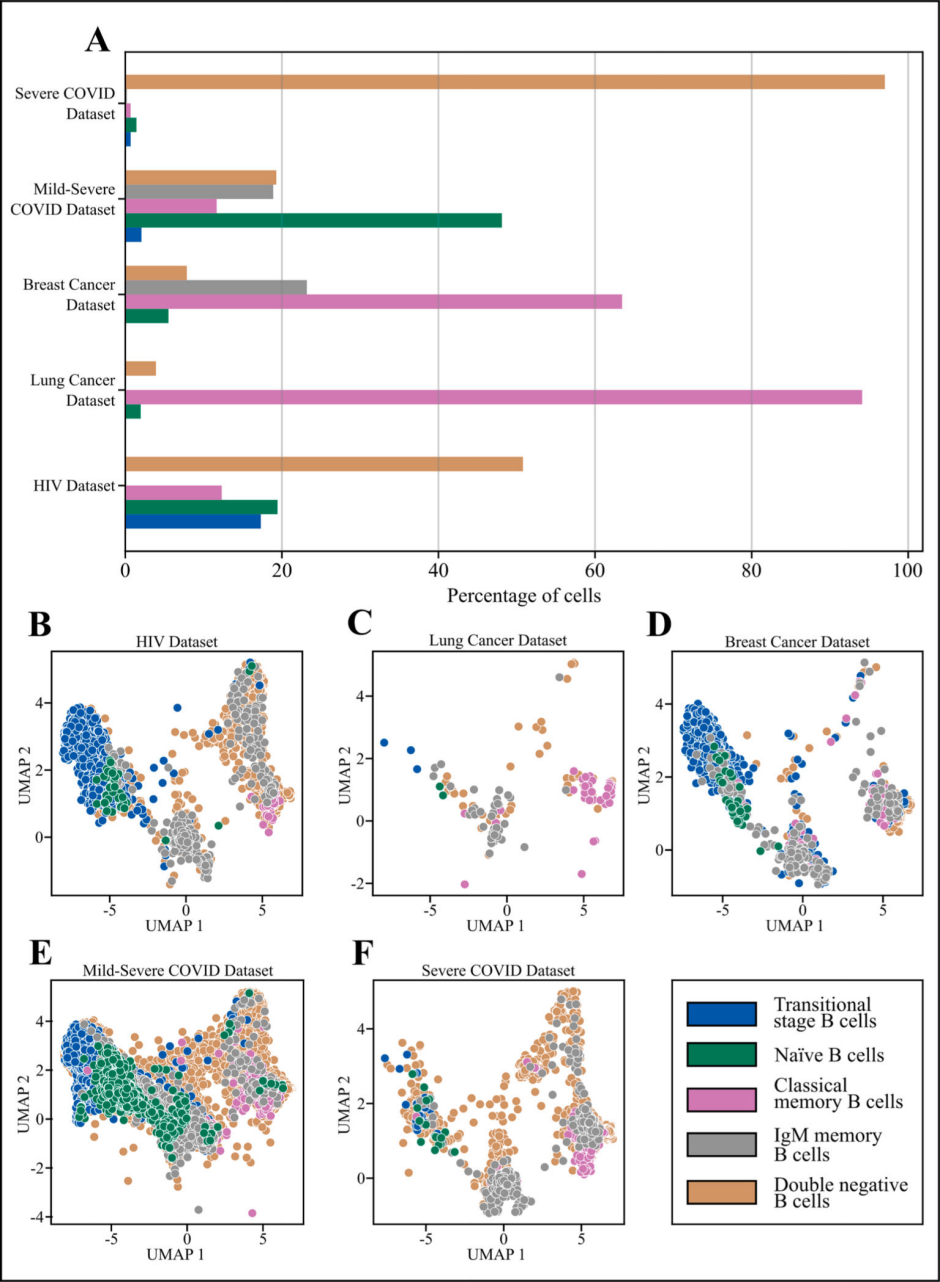
Characterization of B cells across infections and diseases using Azimuth. The percentage of B cells in the HIV dataset (3621 total cells), lung cancer dataset (153 total cells), breast cancer dataset (2356 total cells), mild-severe COVID dataset (25068 total cells), and severe COVID dataset (48491 total cells) that mapped to each of the five Azimuth-inferred B cell subtypes based on their scRNA-seq data.

To annotate B cells based on a reference dataset, we used the Azimuth library in R (Hao et al., 2021). The reference single-cell dataset was downloaded from the BioStudies database (Sarkans et al., 2018) and was based on phenotypically sorted peripheral blood B cells discussed in Stewart et al. (2021). This dataset was then normalized using the modeling framework described in Hafemeister and Satija (2019) and counts were integrated across subjects before creating the Azimuth reference. This reference was then used to annotate all B cells in the five datasets (Fig. 2). We also used a knowledge-driven approach to annotate B cells using well characterized cell surface markers and transcription factors (Morgan and Tergaonkar, 2022; Nutt et al., 2015, 2011; Shaffer et al., 2002), particularly BACH2, IGHG1, and PRDM1 (Table A.2; Fig. A.2). Finally, for each of the five datasets, we identified the percentages of Azimuth-annotated B cells subtypes that mapped to each representative attractor as well as the standard error of the mean Hamming distance between the cells and the attractors. In order to determine if the representative attractors and subtypes of B cells were related, we performed chi-squared tests of independence for each combination of network and dataset that was optimized by scBONITA (Table A.3).

### 2.5 .Evaluation of scBONITA-identified attractors using Pystablemotif

In order to evaluate the attractors identified by scBONITA, we compared those attractors to the ones reached by Pystablemotif (Rozum et al., 2022). In particular, the scBONITA-inferred models for the leukocyte transendothelial migration, regulation of actin cytoskeleton, metabolic, and hypoxia signaling pathways were simulated using the Pystablemotif attractor-identification algorithm (max_simulate_size, a parameter that defines the number of variables for which to build a state transition graph, was set in the range of 5–20, ensuring convergence). Finally, the overlapping attractors identified by both scBONITA and Pystablemotif were counted and Hamming distances between the non-overlapping attractors were calculated. Step-by-step analysis code is provided here: https://github.com/Thakar-Lab/scBONITA_attractor_analysis.

## 3. Results & Discussion

### 3.1. B cells scRNA-sequencing datasets summary

In order to investigate signaling cascades underlying B cell differentiation and migration, we curated five different studies from GEO. The datasets are referred to as the HIV study (GSE157966), the Lung Cancer (LC) study (GSE84789), the Breast Cancer (BC) study (GSE135710), the Mild-Severe COVID (MSC) study (GSE164381), and the Severe COVID (SC) study (GSE158038). In brief, the HIV study isolated B cells (CD19+CD20+CD10−) from the peripheral blood of three HIV-infected as well as three healthy adults. This study identified atypical B cells (ABCs) hypothesized to arise in malaria and autoimmune diseases. The expansion of ABCs in these diseases was found to be driven by interferon-γ (Holla et al., 2021). The Lung Cancer study isolated B cells (CD45+CD19+) from non-small-cell lung cancer (NSCLC) tumor samples in order to identify distinct immunophenotypes (Lizotte et al., 2016). In the Breast Cancer study, B cells (CD45+CD19+) were isolated from biopsy samples of four patients with breast cancer. These cells were obtained from patients both before neoadjuvant chemotherapy (BNACT) and after neoadjuvant chemotherapy (ANACT). B cells with strong ICOSL and CR2 expression, together with low IL-10 expression predicted better chemotherapy efficacy (Lu et al., 2020). In the Mild-Severe COVID dataset, CD19+CD38^hi^CD27^hi^ B cells were sorted from peripheral blood. B cells in this dataset were obtained from four healthy subjects and seven subjects with mild COVID-19 who showed few symptoms and were not hospitalized. B cells were also obtained from three COVID-19 subjects with severe disease. This study showed that subjects with mild COVID-19 contained more clonally diverse B cells compared to subjects with severe COVID-19 (Hoehn et al., 2021). Finally, the Severe COVID-19 study isolated B cells by Human Pan-B cell immunomagnetic negative selection kit (STEMCELL Technologies, Cambridge, MA) from the peripheral blood of four healthy adults and thirteen COVID-19 subjects with severe disease. This study showed that in severe COVID-19 patients, SARS-CoV-2 triggers a chronic immune reaction that is primarily driven by TGF-β and that distracts the immune response from the COVID-19 infection (Ferreira-Gomes et al., 2021).

### 3.2 Phenotyping B cells across disease and infection

To uniformly characterize B cells across different datasets we used an unbiased mapping using Azimuth (Hao et al., 2021) and well-defined B cell reference characterizing peripheral B cell subsets (Stewart et al. 2021). B cells were mapped into five subtypes: classical memory B cells, double negative B cells, IgM memory B cells, Naïve B cells, and Transitional B cells (Fig. 2). The prediction and mapping scores for these annotations were quantified in supplementary Fig. A.3. In the HIV dataset, 50.79% of B cells were double negative memory B cells and the second most abundant population was naïve memory B cells (19.44%). In the Lung Cancer dataset, 94.12% of B cells were classical memory B cells. In the Breast Cancer dataset, the top two most abundant B cells were classical memory B cells (63.46%) and IgM memory B cells (23.17%). In the Mild-Severe COVID dataset, the most abundant B cells were naïve B cells (48.11%) and IgM memory B cells (18.89%). >60% of naïve B cells expressed BACH2 and IRF4. Lastly, in the Severe COVID dataset 97.01% of B cells were double negative memory B cells.

In order to further characterize the Azimuth assigned B cell subtypes described above, the expression of cell surface markers and transcription factors were evaluated (Morgan and Tergaonkar, 2022; Nutt et al., 2015, 2011; Shaffer et al., 2002) (Table A.2 and Fig. A.2). Here we describe the expression in more than 60% of the cells in each cluster. In Breast cancer dataset, classical memory B cells were BACH2+ and IRF4+ and IgM memory B cells were IRF4+. In this dataset, Double negative B cells were BACH2+, PRDM1+ and IRF4+. In HIV dataset, two most abundant cell-states did not express any specific transcription factor in >60% of the cells. In the severe COVID dataset, Double negative B cells were PRDM1+ and IRF4+. In the Mild-Severe COVID dataset Naïve B cells were IRF4+ and BACH2+. In the Lung Cancer dataset classical memory B cells were IGHG1+.

Thus, Azimuth assigned memory B cells does not always show an expected pattern of cell surface marker and transcription factor expression. However, Double negative and Naïve B cells showed expected expression of BACH2 (Table A.2).

### 3.3 Discrete state modeling of B cells by scBONITA

**Fig. 3.**
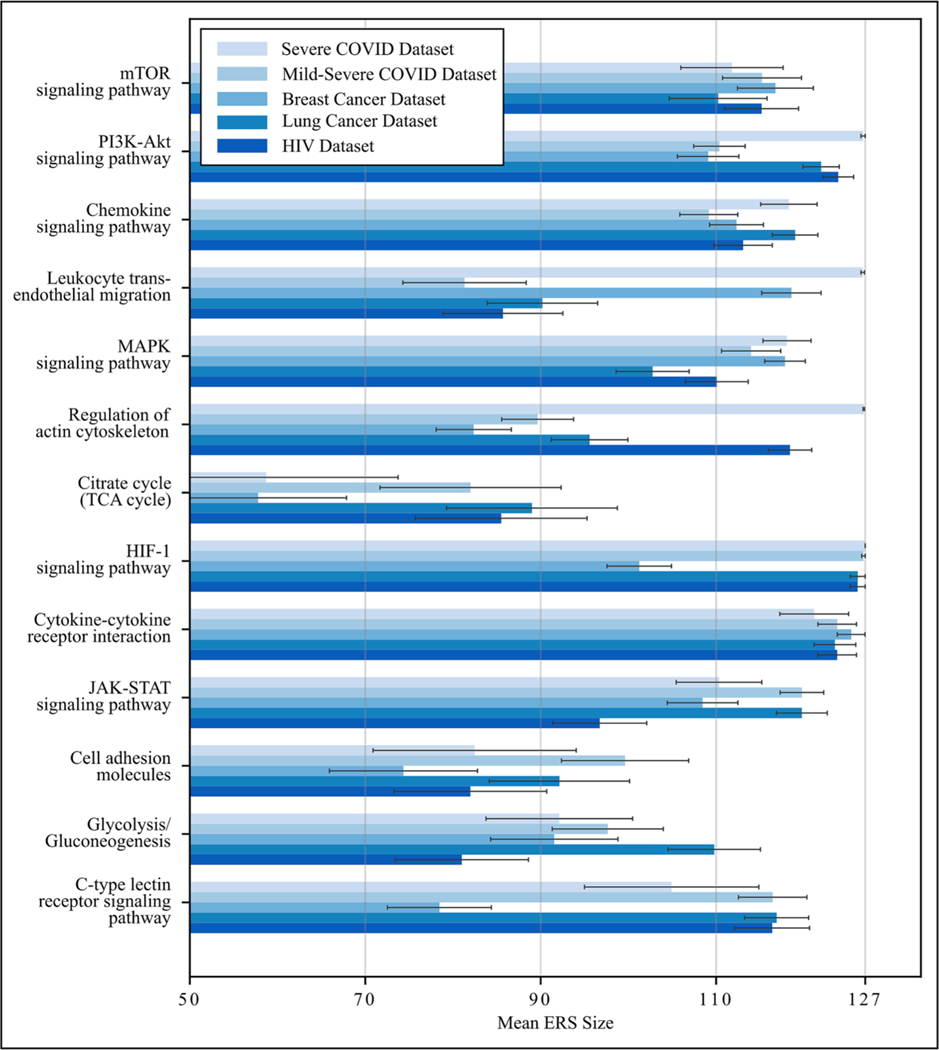
scBONITA-inferred equivalent rule sets (ERS) size (mean ± SEM (Standard Error of Mean)) for all networks across datasets. Networks that were not optimized by scBONITA are not shown.

To investigate the signaling cascades driving B cell differentiation, especially in response to O_2_ levels, we selected 19 pathways based on our prior work (Hilchey et al., 2022, 2020). The discrete-state models for these pathways were learned using scBONITA. The pathways were analyzed separately because they function across various sites such as blood, lymph node that are spatially and temporally resolved with different O_2_ levels. Briefly, scBONITA infers logic rules to depict how upstream regulators control the downstream genes (see methods for details). scBONITA returned optimized Boolean logic rule sets, called Equivalent Rule Sets (ERS). We chose the simplest minimal rule models that maximized the usage of “OR” interactions between genes. Interestingly, not all minimal models were trivial, rather we found models that included “AND” interactions between genes (Table A.4). These regulatory interactions provide rules defining signal integration. A pathway model is considered optimized when the ERS size is less than the maximum number of possible logic rules. Here, we evaluate the ERS sizes of the most complex nodes with three upstream regulators, and which are expected to have a maximum ERS size of 127. Thirteen networks were optimized by scBONITA in at least one of the five datasets (Fig. 3). We conducted further attractor analysis for eight networks in which at least one dataset had an average ERS size less than 100 for nodes with in-degree three: Leukocyte *trans*-endothelial migration pathway, regulation of actin cytoskeleton pathway, cell adhesion molecules pathway, HIF-1 pathway, citrate cycle pathway, glycolysis/gluconeogenesis pathway, JAK-STAT pathway, and c-type lectin receptor pathway. Out of these eight pathways, we will discuss five pathways because their attractors most significantly distinguished disease/infection and healthy states. It is worth noting that none of the eight pathways identified attractors that could distinguish B cells’ diseased and healthy states in the Lung Cancer study because this study sequenced very few cells. Additionally, none of the pathways were optimized by the Severe COVID (SC) dataset. In the following sections we only describe the two attractors that distinguish disease/infection and healthy states and that map to the highest number of cells.

### 3.4 Leukocyte transendothelial migration (LTM) signaling pathway states in B cells

**Fig. 4.**
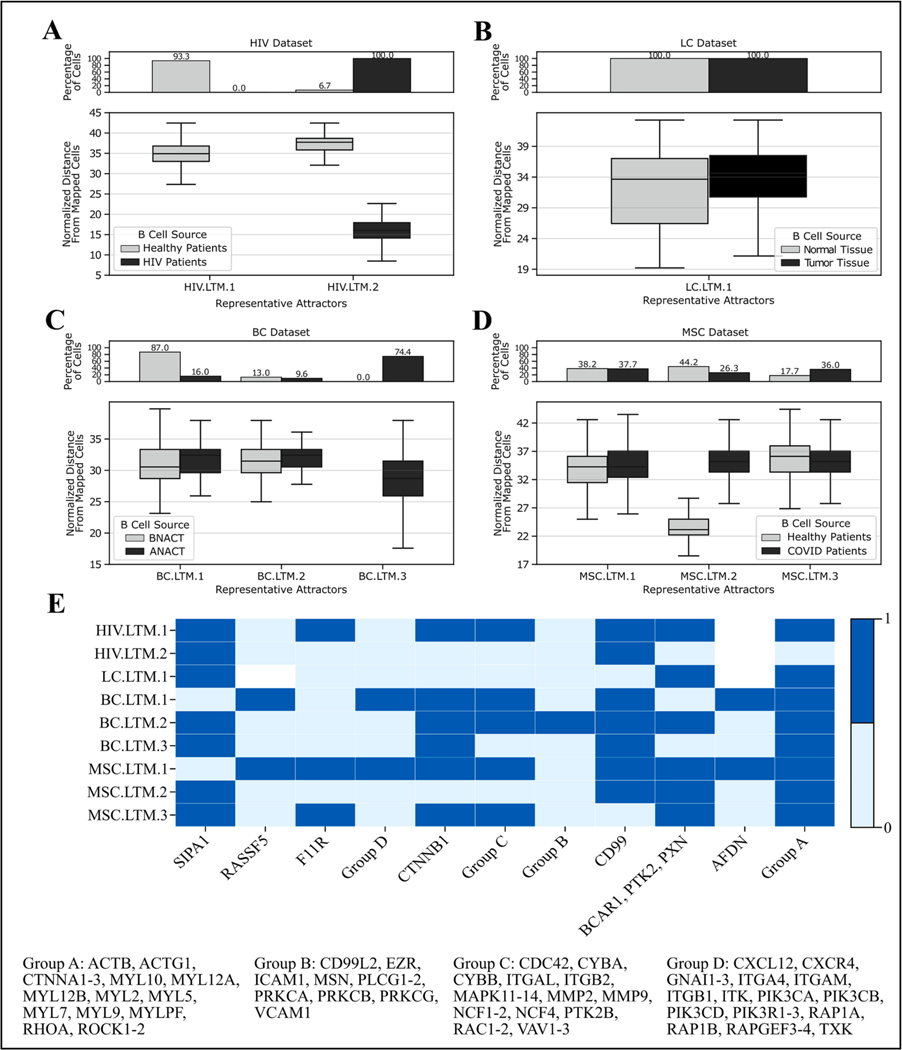
The characterization of B cells using the leukocyte transendothelial migration (LTM) signaling pathway. The correlation (upper panels, bar plots) as well as Hamming distances (lower panels, box plots) between identified representative attractors and B cells source/phenotypes are depicted for B cells obtained from the (A) HIV dataset (B) Lung cancer dataset (C) Breast cancer dataset and (D) Mild-Severe COVID dataset. (E) The gene activation pattern that differed across representative attractors out of the 136 nodes in the network. White squares correspond to genes that were not detected in a dataset. Refer to the Table A.6 for a complete list of genes and their activations states.

Learned regulatory rules for the LTM pathway were simulated to identify attractors that defined the cellular state with respect to LTM (Fig. 4). We identified attractors that significantly mapped to disease/infection or healthy states in the HIV study (chi-squared test, p < .0001), the Breast Cancer study (chi-squared test, p < .0001), and the Mild-Severe COVID study (chi-squared test, p < .0001).

In the HIV study, HIV.LTM.1 mapped to 93.3% of B cells obtained from healthy individuals whereas HIV.LTM.2 mapped to all B cells obtained from HIV patients (Fig. 4A). HIV.LTM.2 was defined by an OFF state of thirteen genes that encode actin, myosin, or catenin proteins important in B cell antigen extraction (Group A in Fig. 4E), suggesting that HIV antigens are invisible to B cells. In contrast, the ON state of those genes in HIV.LTM.1 is likely reflecting healthy B cells that are undergoing surveillance (Wang and Hammer, 2020). There were other well characterized genes activated in HIV.LTM.1 but not in HIV.LTM.2, including three RhoA-ROCK pathway genes in Group A that are involved in B cell development and activation (Ricker et al., 2016) as well as genes in Group C that are important for the activation, survival, and migration of B cells, including Cdc42 (Gerasimcik et al., 2015), ITGB2 (Nagy-Baló et al., 2020), PTK2B (Tse et al., 2009), Rac genes (Walmsley et al., 2003), VAV genes (Malhotra et al., 2009), and the MAPK pathway genes. Since the above genes are essential for B cells’ viability and function, we hypothesize that the OFF state of those genes in HIV.LTM.2 resembles the state of B cell exhaustion that is common in patients with HIV as reviewed in Moir and Fauci (2014).

In the Breast Cancer study, BC.LTM.1 mapped to 87.0% of B cells obtained from BC tissue of BNACT patients. In comparison, BC.LTM.3 mapped to 74.4% of B cells obtained from BC tissue of ANACT patients (Fig. 4C). When investigating the pattern of gene activation that define each attractor, BC.LTM.1 and BC.LTM.3 differed in the activation of several key groups of genes. Most notable are CXCL12 and CXCR4 (Group D in Fig. 4E) which promote angiogenesis and tumorigenesis (Zheng et al., 2007; Zielińska and Katanaev, 2020), as well as regulate O_2_ mediated cascades. Since these genes are OFF in BC.LTM.3, the analysis suggests that neoadjuvant chemotherapy inhibits tumorigenic and angiogenetic effects. Additionally, the activation of the following key genes differed in BC.LTM.1 and BC.LTM.3: Group C genes mentioned previously, Group D genes such as GNAI loss of which eliminates B lymphocyte compartments (Hwang et al., 2013) as well as PIK3 and RAP genes that promote B cell development, survival, and trafficking (Chen et al., 2008). The role of B cells in cancer is complex with either positive, negative, or no effects on tumor growth depending on their location (Laumont et al., 2022; Yuen et al., 2016). Since the aforementioned genes in Group C and Group D promote B cells viability and function, the B cell state in which those genes are OFF, as represented by BC.LTM.3, suggests that neoadjuvant chemotherapy reduces the activation of B cells in response to tumor antigen.

Lastly, in the Mild-Severe COVID study, MSC.LTM.2 and MSC.LTM.3 significantly mapped to B cells obtained from healthy and COVID-infected patients respectively (Fig. 4D). When investigating the pattern of gene activation that define each attractor, Group C genes were ON in MSC.LTM.3 but not in MSC.LTM.2 (Fig. 4E). Given the importance of these genes in B cell activation, we hypothesize that MSC.LTM.3 reflects B cells’ activation, and therefore highlights genes that could play important roles in the acute respiratory distress syndrome (ARDS) and the cytokine storm, to which the majority of deaths in COVID-19 patients are attributed (Fara et al., 2020). In fact, the MAPK pathway genes have already been identified as drug targets for patients with COVID-19 (Huang et al., 2020).

### 3.5 Regulation of actin cytoskeleton (RAC) signaling pathway states in B cells

**Fig. 5.**
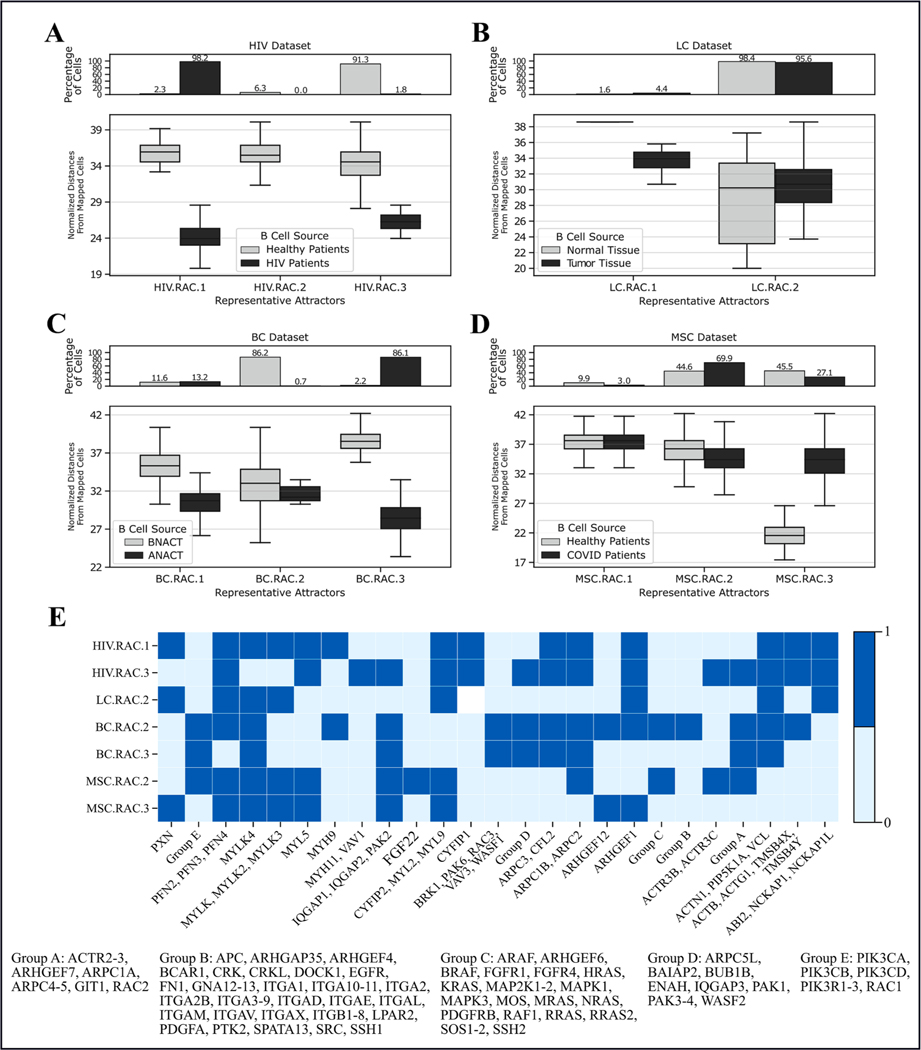
The characterization of B cells using the regulation of actin cytoskeleton (RAC) signaling pathway. The correlation (upper panels, bar plots) as well as Hamming distances (lower panels, box plots) between identified representative attractors and B cells source/phenotypes are depicted for B cells obtained from the (A) HIV dataset (B) Lung cancer dataset (C) Breast cancer dataset and (D) Mild-Severe COVID dataset. (E) The gene activation pattern that differed across representative attractors out of the 256 nodes in the network. White squares correspond to genes that were not detected in a dataset. Refer to the Table A.7 for a complete list of genes and their activations states.

Attractors of the RAC signaling pathway significantly mapped to B cells from healthy and diseased/infected patients for B cells obtained from the HIV (chi-squared test, p < .0001), Breast Cancer (chi-squared test, p < .0001), and Mild-Severe COVID (chi-squared test, p < .0001) datasets (Fig. 5).

In the HIV study, HIV.RAC.3 mapped to 91.3% of B cells from healthy subjects while HIV.RAC.1 mapped to 98.2% of B cells obtained from HIV infected subjects (Fig. 5A). Key genes such as RAC2 and VAV1, which were also identified in the LTM signaling pathway, had an ON state in HIV.RAC.3 but an OFF state in HIV.RAC.1 (Fig. 5E). Similarly, other genes involved in B cell activation and trafficking were ON in HIV. RAC.3 but OFF in HIV.RAC.1, including the ARPC5L and PAK genes (Group D in Fig. 5E) as well as genes that encode actin proteins or components of the Arp2/3 complex (Group A in Fig. 5E) (Bolger-Munro et al., 2019; Grebeñová et al., 2019´). The OFF state of those genes in HIV.RAC.1 resembles B cells exhaustion in HIV positive subjects, which is reviewed in Moir and Fauci (2014).

In the Breast Cancer study, BC.RAC.2 mapped to 86.2% of B cells taken from BNACT patients while BC.RAC.3 mapped to 86.1% of B cells obtained from ANACT patients (Fig. 5C). Group B and Group C genes were ON in BC.RAC.2 but OFF in BC.RAC.3 (Fig. 5E). Group B included several integrin genes important for B cell activation and trafficking. Group C included genes involved in the Ras/Raf/MAPK pathway, which has been implicated in B cell proliferation, differentiation, and apoptosis. The inactive state of those genes in BC.RAC.3 likely signifies the reduction of B cells trafficking and activation in breast cancer tissue ANACT.

In the Mild-Severe COVID study, MSC.RAC.2 and MSC.RAC.3 significantly mapped to B cells taken from COVID-infected and healthy subjects respectively (Fig. 5D). Several key genes were active in MSC. RAC.2 but inactive in MSC.RAC.3 (Fig. 5E) including ARHGEF7, actin genes, and genes that encode components of the Arp2/3 complex (Group A in Fig. 5E). Similarly, genes in Group C were active in MSC.RAC.2 but not in MSC.RAC.3, including the Ras/Raf/MAPK pathway genes as well as the FGF genes, which are considered as drug targets in patients with COVID-19 (Cuevas and Manquillo, 2020; Huang et al., 2020). MSC. RAC.2 also represented an active state for Group E genes which included the PI3K signaling pathway genes, often over activated in patients with COVID-19 and used as drug targets for the prevention of coagulation complications (Khezri et al., 2022).

### 3.6 Metabolic and hypoxia signaling pathway states in B cells

HIF1A is a prominent factor involved in regulating cellular processes in response to hypoxic conditions. For instance, in cancer cells, HIF1A promotes the activation of its downstream proangiogenic genes, which in turn support tumorigenesis. HIF1A also promotes downstream genes that inhibit TCA cycle metabolism and promote anerobic metabolism, a process often referred to in cancer studies as the Warburg effect (Courtnay et al., 2015). While this effect is established in cancer cells, its role on B cells viability and function is still largely unknown. Indeed B cells in cancer tissue are exposed to the hypoxic environment.

**Fig. 6.**
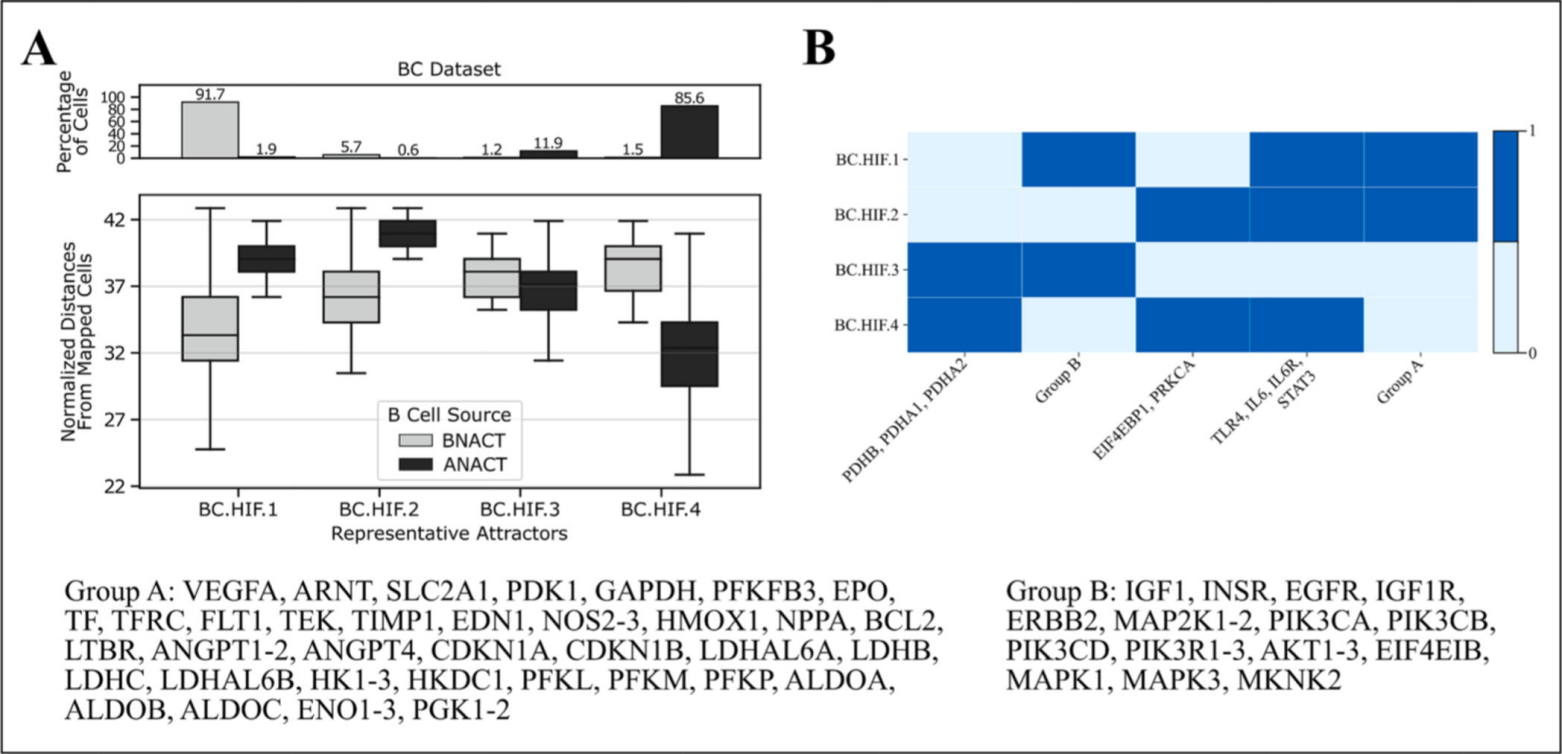
The characterization of B cells using the HIF1A (HIF) signaling pathway. (A) The correlation (upper panel) as well as Hamming distances (lower panel) between identified representative attractors and B cells source/phenotypes are depicted for B cells obtained from the breast cancer dataset. (E) The gene activation pattern that differed across representative attractors out of the 159 nodes in the network. Refer to the Table A.8 for a complete list of genes and their activations states.

The HIF1A (HIF) pathway was only optimized in the Breast Cancer study, where the attractors BC.HIF.1 and BC.HIF.4 significantly mapped (chi-squared test, p < .0001) to 91.7% and 85.6% of the B cells obtained from BNACT and ANACT patients respectively (Fig. 6A). Given the activation pattern of BC.HIF.1 (Fig. 6B), we hypothesize that this attractor represents a state in which HIF-1 is activated along with its downstream proangiogenic genes shown in Group A, such as VEGF, TIMP1, FLT1, and ANGPT. In Group A, the genes TF, TFRC, and EPO are also active in BC.HIF.1. As these genes are involved in erythropoiesis, iron metabolism, and thus oxygen delivery to cancer tissue (Chan et al., 2017), the active state of those genes in BC.HIF.1 supports its resemblance to an angiogenesis and tumorigenesis promoting state. In contrast, neoadjuvant chemotherapy eliminated the proangiogenic state as depicted by the inactivity of Group A genes in BC.HIF.4. Moreover, PI3K/ACT and MAPK pathways’ genes in Group B are inactive in BC. HIF.4 but active in BC.HIF.1 (Fig. 6B). These genes are important in B cell development and survival (Abdelrasoul et al., 2018), hence their inactive state in BC.HIF.4 likely signifies a decrease in the activation of B cells in response to tumor antigen in breast cancer tissue post- neoadjuvant chemotherapy.

As previously mentioned, HIF1A also influences the metabolic state of cells. The differences in gene activity between BC.HIF.1 (associated with BNACT status) and BC.HIF.4 (associated with ANACT status) indicate a post-treatment metabolic shift from primarily glycolysis followed by lactic acid fermentation to glycolysis followed by TCA cycle activity and oxidative phosphorylation. This is shown by the differential activity of genes in the two attractors. BC.HIF.1 has ‘ON’ activity of PDK1, which has been shown to inhibit pyruvate dehydrogenase in a HIF-1 dependent manner (Kim et al., 2006). In addition, BC.HIF.1 has ‘ON’ activity of the glucose transporter SLC2A1, the glycolysis enzymes PFKB3, ALDOA, PFKL, ENO1 and the lactic dehydrogenase LDHA. These genes, which are also downstream of HIF1A, were inactive in BC.HIF.4, (Dupuy et al., 2015). This metabolic shift was also noted when we investigated the attractors identified in the citrate cycle (CC) signaling pathway in the Breast Cancer study. In particular, BC.CC.1, an attractor in which 82.4% of CC genes were inactive, mapped to the majority (95.8%) of B cells taken from the BNACT group. Conversely, BC.CC.2, an attractor in which these CC genes were active, mapped to 57.3% of B cells taken from the ANACT group (Fig. A.4). These differences in gene activity coupled with the differential attractor assignment between BNACT and ANACT provide evidence for a post-treatment disruption of the tumor microenvironment leading to a return to normal cellular metabolism.

### 3.7 B cell subtype specific attractor mapping

We showed that the B cell pathway specific attractors mapped to healthy vs disease/infection states. Next, we wanted to evaluate whether these pathway specific attractors define B cell subtypes. To perform this analysis, we used Azimuth (Hao et al., 2021) along with a Stewart et al. (2021) reference dataset to annotate cells from all five datasets (Fig. 2). ln five pathways, chi-square tests showed that there was a significant relation between the representative attractors and the Azimuth- annotated subtypes of B cells (Table A.3).

**Fig. 7.**
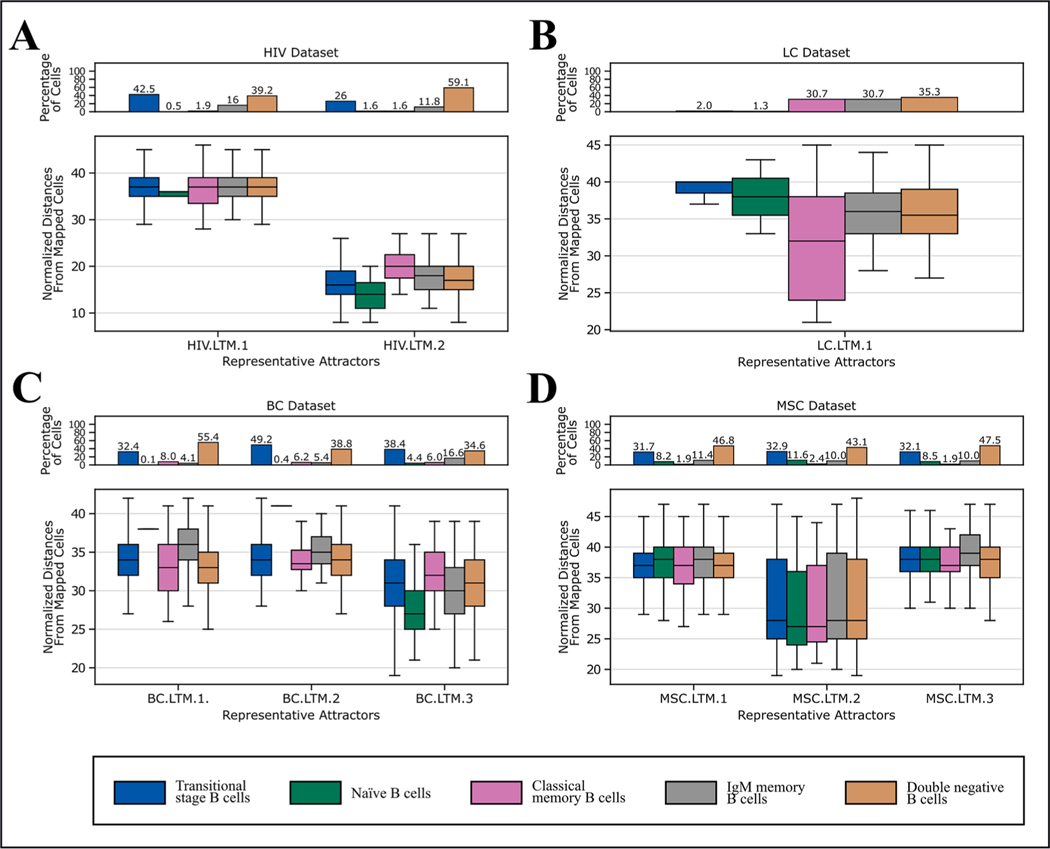
The leukocyte transendothelial migration (LTM) signaling pathway attractors mapping to the Azimuth-inferred B cell subtypes. The distribution (upper panels, bar plots) and Hamming distances (lower panels, box plots) of Azimuth-inferred B cell subtypes across representative attractors for B cells obtained from the (A) HIV dataset (B) Lung cancer dataset (C) Breast cancer dataset and (D) Mild-Severe COVID dataset.

**Fig. 8.**
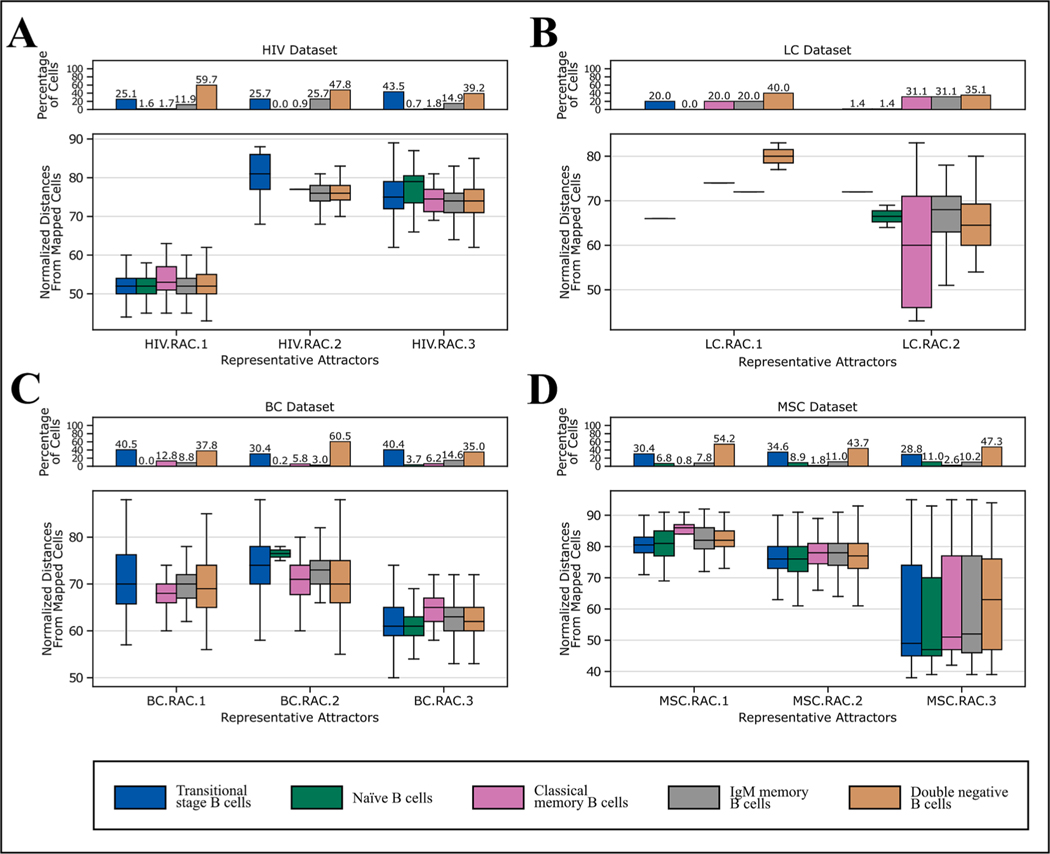
The regulation of actin cytoskeleton (RAC) signaling pathway attractors mapping to the Azimuth-inferred B cell subtypes. The distribution (upper panels, bar plots) and Hamming distances (lower panels, box plots) of Azimuth-inferred B cell subtypes across representative attractors for B cells obtained from the (A) HIV dataset (B) Lung cancer dataset (C) Breast cancer dataset and (D) Mild-Severe COVID dataset.

In the HIV study, there was a 38.8% and 26.25% decrease in the percentage of transitional stage B cells and IgM memory B cells respectively in HIV.LTM.2 in comparison to HIV.LTM.1 but a 50.8% increase in the percentage of double negative memory B cells (chi- squared test, p < .0001), which could also be ascribed the role of atypical memory B cells (Fig. 7A). The expansion of atypical memory B cells is a common feature in HIV patients that was also noted in the original HIV study (Holla et al., 2021). Specifically, this expansion has been attributed to chronic immune activation and inflammation, and it has been implicated in the reduced acquisition of immunity in chronic infectious diseases (Portugal et al., 2017). This same expansion has also been observed when investigating the HIV dataset through other signaling pathways including RAC (Fig. 8A), CC (Fig. A.6), and Glycolysis/Gluconeogenesis (GG) (Fig. A.7), where the percentage of double negative memory B cells increased by 52.3%, 55.9%, and 38.4% respectively in each pathway’s attractor that was mapping to B cells obtained from HIV patients in comparison to the attractor mapping to B cells obtained from healthy patients.

**Fig. 9.**
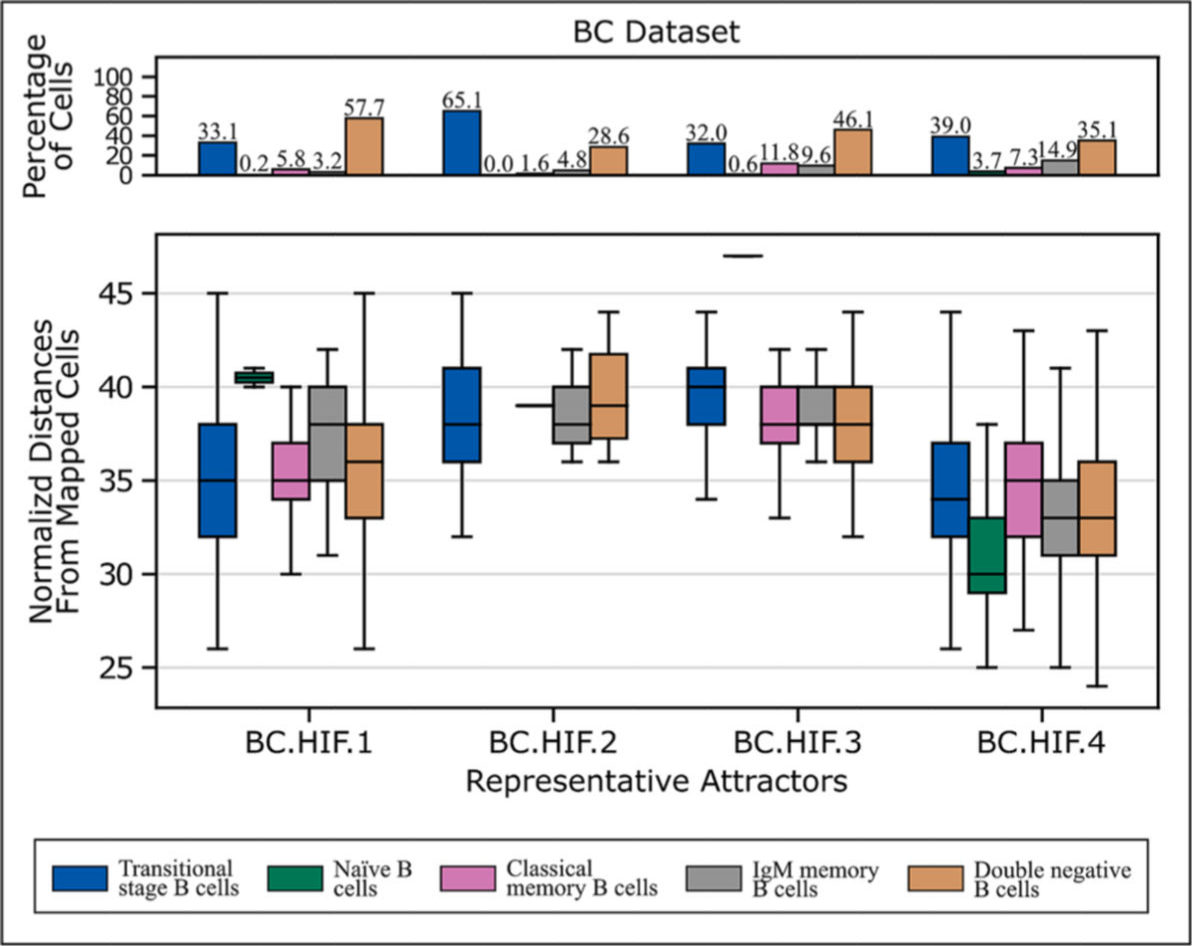
The HIF1A (HIF) signaling pathway attractors mapping to the Azimuth-inferred B cell subtypes. The distribution (upper panel) and Hamming distances (lower panel) of Azimuth-inferred B cell subtypes across representative attractors for B cells obtained from the breast cancer dataset.

In contrast, when investigating B cells obtained from the Breast Cancer dataset, the LTM (Fig. 7C), RAC (Fig. 8C), HIF (Fig. 9), CC (Fig. A.6), and GG (Fig. A.7) signaling pathways showed a 37.5%, 42.1%, 39.2%, 33.9%, and 36.3% decrease in the percentage of double negative memory B cell respectively in each pathway’s attractor that was mapping to B cells obtained after neoadjuvant chemotherapy in comparison to the attractor mapping to B cells obtained prior to neoadjuvant chemotherapy. This decrease in the percentage of double negative memory B cells was matched by an increase in the percentage of transitional stage B cells or/and IgM memory B cells.

### 3.8 Evaluation of attractor analysis

To evaluate scBONITA steady state analysis, we compared the attractor analysis presented above with the attractors identified by Pystablemotif library (Rozum et al., 2021), which utilized the algorithm described in (Rozum et al., 2021). Specifically, we used five networks (LTM, RAC, HIF, CC, and GG) and three datasets (HIV, BC, and MSC) for this comparison. Pystablemotif failed to converge and identify attractors for two pathways: RAC and CC. On average, 12.53% (std. Dev = 6.57) of the attractors identified by pystablemotifs were reachable attractors based on their mapping to cells. In conclusion, Pystablemotif characterizes a large repertoire of the attractors, however a very small percentage of that is empirically measured. Furthermore, we found that the attractors identified by scBONITA represented on average 5.43% (std. Dev = 4.37) of the complete attractor repertoire from pystablemotifs. This is expected given that scBONITA only characterizes empirically observed attractors. Thus, these attractors are the biologically relevant steady states or phenotypes of the tested networks. The differences between the attractors are due to different update algorithms which have been shown to impact attractor repertoire (Fauré et al., 2006; Garg et al., 2008).

## 4. Conclusion

**Fig. 10.**
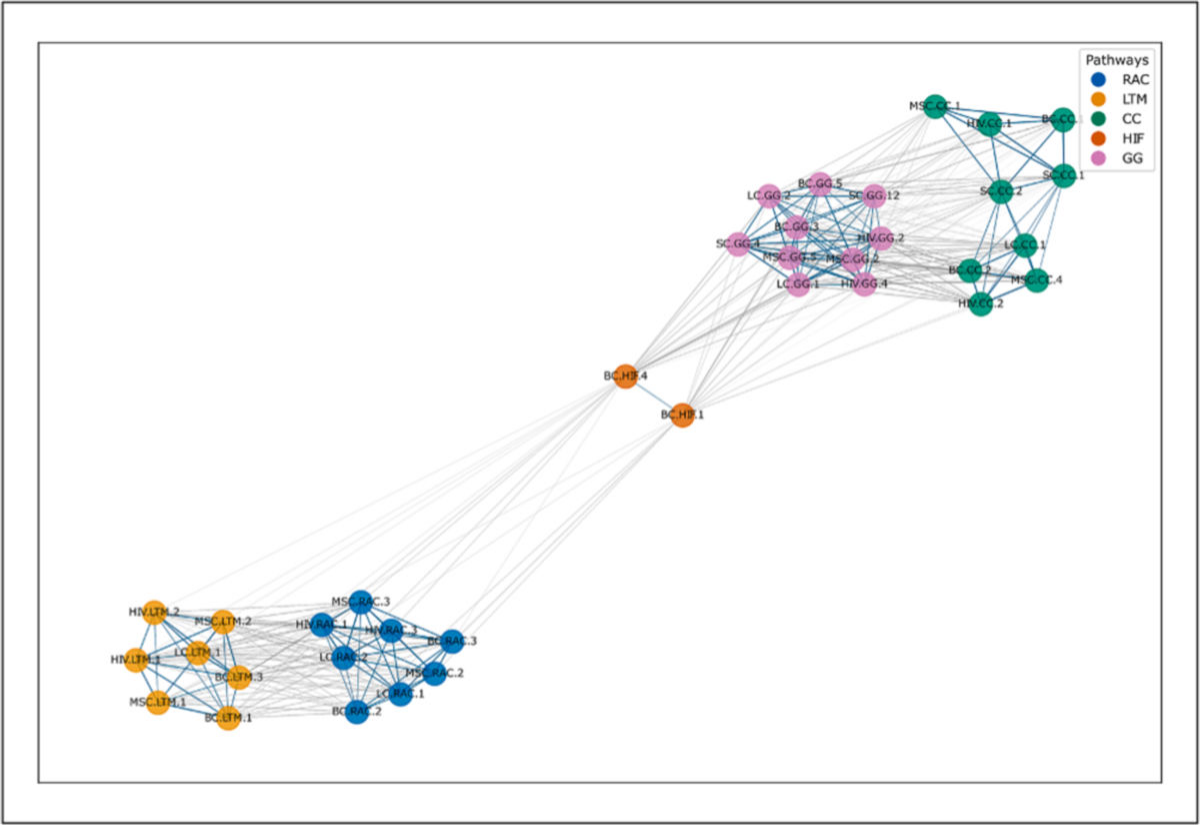
Interactions between pathway-specific attractors: Nodes represent top two pathway attractors for each health status (shown by node name). These nodes were positioned using the NetworkX library spring_layout function which uses the Fruchterman-Reingold force-directed algorithm. Edges connecting pathway-specific attractors indicate the similarity between them and the edges connecting different pathways are weighted by the number of genes in the same state that are overlapping between the pathways. Pathways- CC: Citrate Cycle (blue), HIF: HIF1A pathways (yellow), LTM: Leukocyte Transendothelial Migration (green), RAC: Regulation of Actin Cytoskeleton (red), GG: Glycolysis/Gluconeogenesis (pink).

In this paper, we evaluate the application of Boolean modeling and attractor analysis for classifying sequenced cells based on their pathway- specific signaling states. We show that signaling pathway-specific attractors found using scBONITA and steady state analysis can be used to differentiate B cells’ states in health and disease. The gene activation patterns of these states corresponded to biologically relevant B cell phenotypes in HIV, breast cancer, and COVID-19. Here we evaluated each pathway separately because these pathways are functional in spatially and temporally resolved manner as B cells’ response evolve from the antigen recognition to clonal expansion involving coordinated migration to secondary lymphoid tissues. Secondary lymphoid tissues normally expose B cells to a low O2 (hypoxic) environment compared to blood. Our results show different attractors even across healthy cells from different studies. Although, theoretically there could be finite attractors, in reality various variables such as time of sampling, variations in the reagents, technical limitations in the measurements at the single cell resolution, site of sampling, the immune history of each subject, etc. impact the attractors. Our work shows the sets of genes that are consistent across different health status, which is a first step towards identifying consistent pathway specific attractors. To investigate the relationship between the pathway specific attractors we evaluated the similarity between each pathways specific attractor and the attractors across multiple pathways (Fig. 10). Fig. 10 highlights the central role of HIF1-α pathway in connecting metabolic pathways with migration related pathways.

We note that this method of classifying cells based on pathway states is highly dependent on the quality of the rule inference. The rule inference is limited by the clarity of signal in the training dataset; i.e., if multiple non-consistent signaling states are represented in the dataset. It is then correspondingly harder or impossible to enumerate reachable attractor states. However, scBONITA could resolve the signaling states as shown by the reduced ERS and thus enumerate the reachable attractors. We also note that the quality of cell-to-attractor mapping is highly dependent on several heuristic parameters, notably the clustering/merging of similar attractor states and the similarity thresholds between cells and attractors. One of the limitation of using restricted network topologies and restricting the analysis to isolated signaling pathways is that it ignores the interplay between signaling pathways, which is reflected in the observed cell expression. Larger, well-characterized signaling pathways mitigate this to some extent. However, our findings show the potential of our computational methods in providing valuable information on the relative importance of different signaling components in immune cells, how they influence one another, and how these relationships may vary across conditions. In addition, the gene activation patterns along with importance scores identified by the scBONITA can lay out informed hypotheses for experimental testing of existing and novel pharmaceutical compounds, as well as potential targets for clinical manipulation of the immune response.

## Supplementary Material

MMC5

MMC7

MMC6

MMC4

MMC3

MMC1

MMC2

## Figures and Tables

**Table 1:** Networks used for rule inference and later identification of cellular steady states.

Category	Database	ID	Name
Signal Transduction	KEGG	hsa04010	MAPK signaling pathway
		hsa04370	VEGF signaling pathway
		hsa04630	JAK-STAT signaling pathway
		hsa04668	TNF signaling pathway
		hsa04066	HIF-1 signaling pathway
		hsa04020	Calcium signaling pathway
		hsa04151	PI3K-Akt signaling pathway
		hsa04150	mTOR signaling pathway
Carbohydrate Metabolism	KEGG	hsa00010 hsa00020	Glycolysis/Gluconeogenic Citrate Cycle (TCA Cycle)
Signaling molecules and interaction	KEGG	hsa04060	Cytokine-cytokine receptor interaction
		hsa04512	ECM-receptor interaction
		hsa04514	Cell adhesion molecules
Immune system	KEGG	hsa04670	Leukocyte transendothelial migration
		hsa04625	C-type lectin receptor signaling pathway
		hsa04062	Chemokine signaling pathway
	WikiPathways	WP23	B cell receptor signaling pathway
Folding, sorting and degradation	KEGG	hsa04120	Ubiquitin mediated proteolysis
Cell motility	KEGG	hsa04810	Regulation of actin cytoskeleton
