# Supplementary material for: Discrete-state models identify pathway specific B cell states across diseases and infections at single-cell resolution
Source: J Theor Biol. Author manuscript; Available in PMC 2024 Apr 26. (PMC11046450; doi:10.1016/j.jtbi.2024.111769)
Supplement: MMC1 [file NIHMS1977211-supplement-MMC1.docx]

**Appendix Tables**

| Pathway | Dataset | X | dof | p-value |
| --- | --- | --- | --- | --- |
| Leukocyte Trans-endothelial Migration | HIV Dataset | 3166.49 | 1 | < 0.0001 |
|  | Lung Cancer Dataset | NA | NA | NA |
|  | Breast Cancer Dataset | 1353.03 | 2 | < 0.0001 |
|  | Mild-Severe COVID Dataset | 541.06 | 2 | < 0.0001 |
| Regulation of Actin Cytoskeleton | HIV Dataset | 3331.18 | 2 | < 0.0001 |
|  | Lung Cancer Dataset | 0.27 | 1 | 0.606 |
|  | Breast Cancer Dataset | 1941.49 | 2 | < 0.0001 |
|  | Mild-Severe COVID Dataset | 695.98 | 2 | < 0.0001 |
| Cell Adhesion Molecules | HIV Dataset | 27.17 | 1 | < 0.0001 |
|  | Lung Cancer Dataset | NA | NA | NA |
|  | Breast Cancer Dataset | 2209.55 | 9 | < 0.0001 |
|  | Mild-Severe COVID Dataset | 960.05 | 29 | < 0.0001 |
|  | Severe COVID Dataset | 2634.85 | 10 | < 0.0001 |
| HIF-1 signaling pathway | Breast Cancer Dataset | 2114.48 | 3 | < 0.0001 |
| Citrate Cycle | HIV Dataset | 3224.16 | 3 | < 0.0001 |
|  | Lung Cancer Dataset | NA | NA | NA |
|  | Breast Cancer Dataset | 906.96 | 3 | < 0.0001 |
|  | Mild-Severe COVID Dataset | 680.11 | 3 | < 0.0001 |
|  | Severe COVID Dataset | 1755.78 | 4 | < 0.0001 |
| Glycolysis/Gluconeogenesis | HIV Dataset | 3021.55 | 9 | < 0.0001 |
|  | Lung Cancer Dataset | NA | NA | NA |
|  | Breast Cancer Dataset | 1005.57 | 5 | < 0.0001 |
|  | Mild-Severe COVID Dataset | 102.18 | 9 | < 0.0001 |
|  | Severe COVID Dataset | 2294.67 | 17 | < 0.0001 |
| JAK-STAT signaling pathway | HIV Dataset | 3418.66 | 2 | < 0.0001 |
| C-type Lectin Receptor signaling pathway | Breast Cancer Dataset | 1650.49 | 6 | < 0.0001 |
|  | Severe COVID Dataset | 1849.12 | 5 | < 0.0001 |

Table A.1. Contingency chi-squared tests that examined the relation between each signaling pathway’s representative attractors and the source/phenotype of the B cells in each of the five datasets.

| B cell type | Transcriptional profile |
| --- | --- |
| Not B cells | Expresses zero CD3E, GNLY, CD14, FCER1A, GCGR3A, LYZ, PPBP and CD8A. |
| Naïve B cells | Upregulated: IGHD, IL4R, TCL1A, IGHM, and BACH2. |
| Memory B cells | Upregulated: CD24, TNFRSF13B, *IGHG1-4*, *IGHA1-2*. |
| Atypical B cells | Upregulated: TBX21(Tbet), *FCRL5*, *ITGB*2, ZBTB32, NR4A2, TOX2.  Downregulated: TCF7 |
| Plasma B cells | Upregulated: *PRDM1(Blimp-1), XBP1, IRF4*, and *MYC*  Downregulated: PAX5 |

Table A.2. The transcriptional markers used to categorize B cell into subtypes. The markers’ regulation in the different subtypes of B cells was based on their transcriptional expression as established in literature.

| Pathway | Dataset | p-value | X | dof |
| --- | --- | --- | --- | --- |
| Leukocyte Trans-endothelial Migration | HIV Dataset | < 0.0001 | 164.58 | 4 |
|  | Lung Cancer Dataset | NA | NA | NA |
|  | Breast Cancer Dataset | < 0.0001 | 213.71 | 8 |
|  | Mild-Severe COVID Dataset | < 0.0001 | 42.60 | 8 |
| Regulation of Actin Cytoskeleton | HIV Dataset | < 0.0001 | 189.68 | 8 |
|  | Lung Cancer Dataset | 0.0595 | 9.07 | 4 |
|  | Breast Cancer Dataset | < 0.0001 | 212.16 | 8 |
|  | Mild-Severe COVID Dataset | < 0.0001 | 76.29 | 8 |
| HIF-1 signaling pathway | Breast Cancer Dataset | < 0.0001 | 200.81 | 12 |
| Citrate Cycle | HIV Dataset | < 0.0001 | 199.69 | 12 |
|  | Lung Cancer Dataset | NA | NA | NA |
|  | Breast Cancer Dataset | < 0.0001 | 245.68 | 12 |
|  | Mild-Severe COVID Dataset | < 0.0001 | 61.22 | 12 |
|  | Severe COVID Dataset | < 0.0001 | 92.20 | 12 |
| Glycolysis/  Gluconeogenesis | HIV Dataset | < 0.0001 | 104.15 | 12 |
|  | Lung Cancer Dataset | 0.6862 | 2.27 | 4 |
|  | Breast Cancer Dataset | < 0.0001 | 233.72 | 12 |
|  | Mild-Severe COVID Dataset | < 0.0001 | 206.46 | 8 |
|  | Severe COVID Dataset | < 0.0001 | 166.68 | 24 |

Table A.3. Contingency chi-squared tests that examined the relation between each signaling pathway’s representative attractors and the Azimuth-inferred subtypes of the B cells in each of the five datasets.

| Pathway | Dataset | AND regulatory rules |
| --- | --- | --- |
| Leukocyte Trans-endothelial Migration | HIV Dataset | CLDN16 *= (OCLN and ESAM and CLDN7) |
|  | Lung Cancer Dataset | CLDN3 *= (ESAM) or (OCLN and CLDN24)  CLDN7 *= (OCLN and CLDN24) or (CLDN2 and CLDN24)  CLDN23 *= (ESAM) or (OCLN and CLDN24)  CLDN17 *= (ESAM) or (OCLN and CLDN24)  CLDN20 *= (OCLN and CLDN3) or (OCLN and CLDN24)  CLDN22 *= (ESAM) or (OCLN and CLDN24)  CLDN25 *= (ESAM) or (OCLN and CLDN24)  CLDN5 *= (ESAM) or (OCLN and CLDN24)  CLDN8 *= (OCLN and CLDN24) or (CLDN15 and CLDN24)  CLDN6 *= (ESAM) or (OCLN and CLDN24)  CLDN9 *= (ESAM) or (OCLN and CLDN24) |
| Regulation of Actin Cytoskeleton | Breast Cancer Dataset | LIMK1 *= (PAK1 and ROCK2) or (BUB1B)  ARHGEF7 *= (PAK4 and PAK1) or (BUB1B and PAK4) or (BUB1B and PAK1) |
|  | Mild-Severe COVID Dataset | VCL *= (PIP5K1A and PIP5K1B) or (PIP5K1A and PIP4K2A) |
| Citrate Cycle | Breast Cancer Dataset | IDH1 *= (ACO2 and IDH3B) or (ACO1 and IDH3B)  POR *= (DLAT) or (PC and PDHB) |
|  | Mild-Severe COVID Dataset | IDH3G *= (IDH2 and IDH1) |
| Glycolysis/  Gluconeogenesis | HIV Dataset | GALM *= (HK2 and GCK) or (HK2 and HK1)  G6PC *= (GALM and GPI) or (PGM2 and GALM)  MINPP1 *= (BPGM and PGAM2) or (BPGM and PGAM1)  GPI *= (PFKM and ADPGK) or (PGM2 and PFKM) or (PGM2 and ADPGK)  GAPDHS *= (TPI1 and ALDOB) or (TPI1 and ALDOA)  HK1 *= (PGM2 and GALM) or (PGM1 and GALM)  HK3 *= (GALM and PGM2) or (GALM and GPI)  HKDC1 *= (GALM and PGM2) or (GALM and GPI)  G6PC2 *= (GALM and PGM2) or (GALM and GPI)  PGK2 *= (BPGM and GAPDHS) |
|  | Lung Cancer Dataset | GAPDHS *= (TPI1 and ALDOB) or (TPI1 and ALDOA)  PGM2 *= (ADPGK and GPI)  PGM1 *= (GPI and ADPGK) |
|  | Breast Cancer Dataset | GALM *= (HK1 and HK2 and GCK)  G6PC *= (GPI and PGM2) or (GALM and PGM2)  GCK *= (PGM1 and GALM) or (PGM2 and GALM)  GAPDHS *= (ALDOC and TPI1) or (ALDOA and TPI1)  PGM2 *= (GPI and ADPGK)  PGM1 *= (GPI and ADPGK)  HK2 *= (GPI and PGM2) or (PGM1 and GPI)  HK3 *= (GPI and PGM2) or (GALM and PGM2)  HKDC1 *= (GPI and PGM2) or (GALM and PGM2)  G6PC2 *= (GPI and PGM2) or (GALM and PGM2)  G6PC3 *= (PGM1 and GPI) or (GALM and PGM1) |
|  | Mild-Severe COVID Dataset | PGM2 *= (ADPGK and GPI) |
|  | Severe COVID Dataset | BPGM *= (ENO1 and PGK1) or (ENO2 and PGK1)  MINPP1 *= (BPGM and PGAM4) or (PGAM1 and PGAM4)  PGAM4 *= (ENO1 and BPGM) or (ENO2 and BPGM) |

Table A.4. AND regulatory rules retained in the minimal rule models across datasets.

| Abbreviation | Definition |
| --- | --- |
| LTM | Leukocyte Transendothelial Migration |
| RAC | Regulation of Actin Cytoskeleton |
| HIF | HIF1A |
| CC | Citrate Cycle |
| GG | Glycolysis/Gluconeogenesis |
| LC | Lung Cancer |
| BC | Breast Cancer |
| MSC | Mild-Severe COVID |
| SC | Severe COVID |
| NACT | Neoadjuvant Chemotherapy |
| ANACT | After Neoadjuvant Chemotherapy |
| BNACT | Before Neoadjuvant Chemotherapy |

Table A.5. Abbreviations table.
