# Supplementary material for: Discrete-state models identify pathway specific B cell states across diseases and infections at single-cell resolution
Source: J Theor Biol. Author manuscript; Available in PMC 2024 Apr 26. (PMC11046450; doi:10.1016/j.jtbi.2024.111769)
Supplement: MMC2 [file NIHMS1977211-supplement-MMC2.docx]

**Appendix Figures**


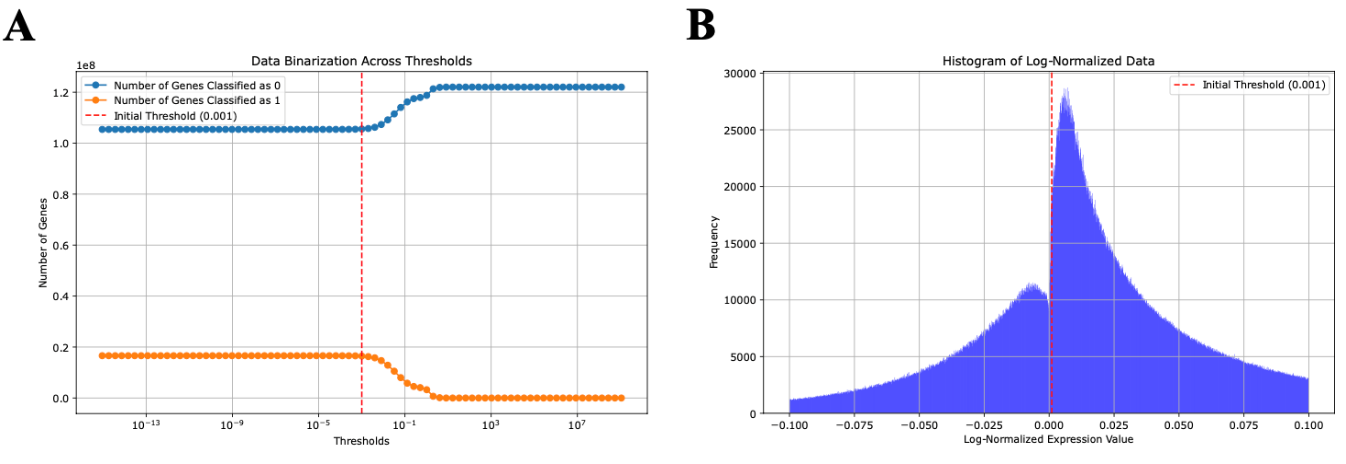


Fig. A.1.


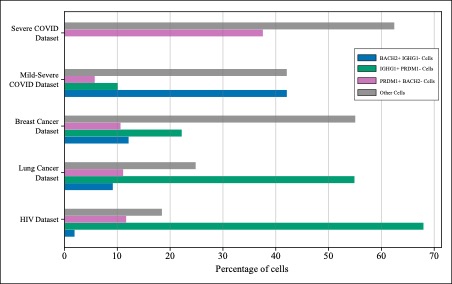


Fig. A.2. The percentage of B cells in each dataset that mapped to each of the five markers-based B cell categories.

Fig. A.3. The distribution of prediction (blue) and mapping (orange) scores shown as split violin plots for all cells reached by Azimuth when inferring the subtype of B cells in each of the five datasets.


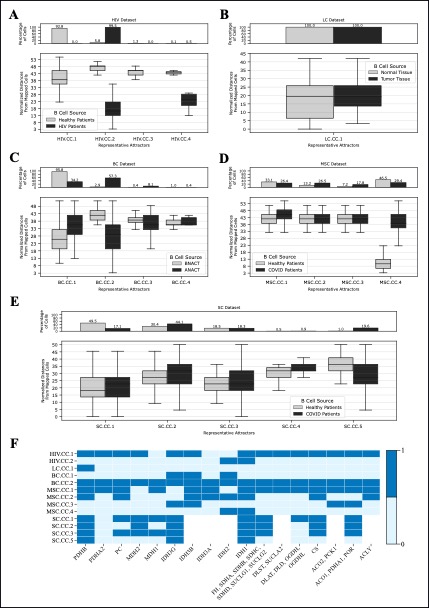


Fig. A.4. The characterization of B cells using the citrate cycle (CC) signaling pathway. The correlation (upper panels, bar plots) as well as Hamming distances (lower panels, box plots) between identified representative attractors and B cells source/phenotypes are depicted for B cells obtained from the (A) HIV dataset (B) Lung cancer dataset (C) Breast cancer dataset (D) Mild-Severe COVID dataset and (E) Severe COVID dataset. (F) The gene activation pattern that differed across representative attractors out of the 118 nodes in the network. White squares correspond to genes that were not detected in a dataset. Refer to the Table A.9 for a complete list of genes and their activations states.


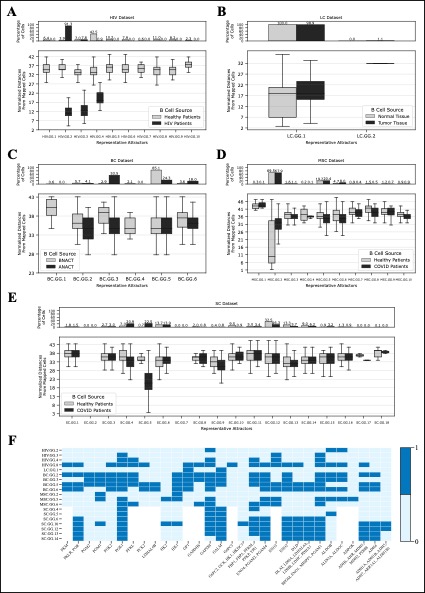


Fig. A.5. The characterization of B cells using the glycolysis/gluconeogenesis (GG) signaling pathway. The correlation (upper panels, bar plots) as well as Hamming distances (lower panels, box plots) between identified representative attractors and B cells source/phenotypes are depicted for B cells obtained from the (A) HIV dataset (B) Lung cancer dataset (C) Breast cancer dataset (D) Mild-Severe COVID dataset and (E) Severe COVID dataset. (F) The gene activation pattern that differed across representative attractors out of the 197 genes in the network. White squares correspond to genes that were not detected in a dataset. Refer to the Table A.10 for a complete list of genes and their activations states.


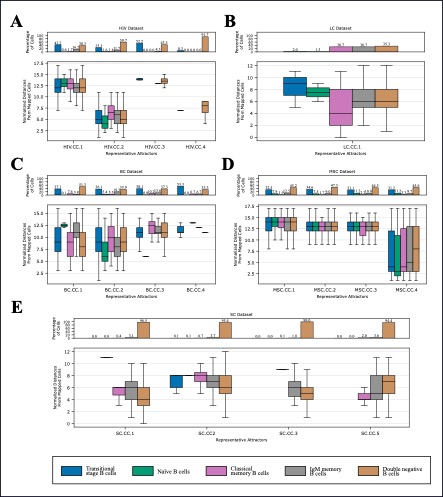


Fig. A.6. The distribution (upper panels, bar plots) and Hamming distances (lower panels, box plots) of Azimuth-inferred B cell subtypes across representative attractors reached using the citrate cycle (CC) signaling pathway for B cells obtained from the (A) HIV dataset (B) Lung cancer dataset (C) Breast cancer dataset (D) Mild-Severe COVID dataset and (E) Severe COVID dataset.


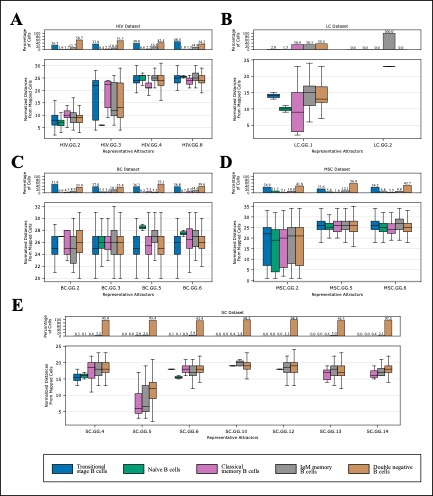


Fig. A.7. The distribution (upper panels, bar plots) and Hamming distances (lower panels, box plots) of Azimuth-inferred B cell subtypes across representative attractors reached using the glycolysis/gluconeogenesis (GG) signaling pathway for B cells obtained from the (A) HIV dataset (B) Lung cancer dataset (C) Breast cancer dataset (D) Mild-Severe COVID dataset and (E) Severe COVID dataset.
